# Exploring blood-based biomarkers in late-life depression: Correlates of psychotherapeutic treatment outcomes

**DOI:** 10.1192/j.eurpsy.2026.10153

**Published:** 2026-01-23

**Authors:** Pamela V. Martino-Adami, Frank Jessen, Frederic Brosseron, Bettina Bewernick, Katharina Domschke, Melanie Luppa, Michael Wagner, Oliver Peters, Lutz Frölich, Steffi Riedel-Heller, Elisabeth Schramm, Alfredo Ramirez, Forugh S. Dafsari

**Affiliations:** 1 Department of Psychiatry and Psychotherapy, University of Cologne, Faculty of Medicine and University Hospital Cologne, Cologne, Germany; 2 German Center for Neurodegenerative Diseases (DZNE), Bonn/Cologne, Germany; 3 Cellular Stress Response in Aging-Associated Diseases (CECAD) Cluster of Excellence, University of Cologne, Faculty of Medicine and University Hospital Cologne, Cologne, Germany; 4 Department of Old Age Psychiatry and Cognitive Disorders, University of Bonn, Bonn, Germany; 5 Department of Psychiatry and Psychotherapy, Medical Center, University of Freiburg, Faculty of Medicine, University of Freiburg, Freiburg, Germany; 6 Institute of Social Medicine, Occupational Health and Public Health, University of Leipzig, Leipzig, Germany; 7 Department of Psychiatry and Psychotherapy, Charité – Universitätsmedizin Berlin, Campus Benjamin Franklin, Berlin, Germany; 8 Department of Geriatric Psychiatry, Central Institute of Mental Health, Medical Faculty Mannheim, University of Heidelberg, Mannheim, Germany; 9 Department of Psychiatry and Glenn Biggs Institute for Alzheimer’s and Neurodegenerative Diseases, 7703 Floyd Curl Drive, San Antonio, Texas, USA

**Keywords:** late-life depression, psychotherapeutic treatment outcome, blood-based biomarkers, cellular senescence, neurodegeneration

## Abstract

**Background:**

Major depressive disorder is a prevalent and debilitating mental health condition contributing to a growing global burden. Late-life depression (LLD), affecting individuals over 60 years of age, is further associated with elevated risks for cardiovascular diseases, cognitive decline, and dementia. Treatment responses vary widely, potentially due to underlying neurodegeneration and cellular senescence. We aimed to explore blood-based biomarkers related to Alzheimer’s disease and senescence-associated secretory phenotype (SASP) proteins, seeking to identify biological underpinnings of LLD and their association with response to psychotherapy.

**Methods:**

We performed a secondary analysis of the Cognitive Behavioral Therapy for Late-Life Depression (CBTlate) trial in 228 participants aged 60 years and older with a diagnosis of LLD. Depression trajectories were compared using clustering. In participants with available plasma samples, biomarker data were generated post hoc. We assessed associations between biomarkers and depression trajectories, biomarker dynamics, and their ability to predict treatment response.

**Results:**

Two depression trajectories were identified: persistently high stable Geriatric Depression Scale (GDS) scores (hsGDS) and decreasing scores over time (dGDS). The hsGDS group had more severe baseline depression (*p* = 2.88 × 10^−6^), anxiety (*p* = 4.39 × 10^−4^), and sleep disorders (*p* = 1.09 × 10^−3^), and was more likely to have a history of major depression (*p* = 0.01) and mild cognitive impairment (*p* = 0.01). Biomarker analysis revealed elevated baseline plasma neurofilament light chain (NfL, *p* = 2.51 × 10^−2^) and reduced C-X-C Motif Chemokine Ligand 5 (CXCL5, *p* = 2.83 × 10^−2^) in the hsGDS group. Including CXCL5 in predictive models improved trajectory differentiation (*p* = 3.94 × 10^−3^).

**Conclusions:**

Cellular aging biomarkers like CXCL5 may improve understanding of LLD and guide personalized therapeutic interventions.

## Introduction

Major depressive disorder (MDD) is a prevalent mental disorder, affecting around 300 million people globally, and is a leading cause of disability [1]. Aging is associated with an increased risk of mental disorders, including MDD, with the clinical presentation and prevalence varying across the lifespan [2]. The trajectory of depression and depressive symptoms varies among individuals [3]. Some may experience clinically significant depressive symptoms only temporarily followed by remission, while others may undergo cycles of remission and relapse, and some may suffer from chronic depression.

Late-life depression (LLD) refers to MDD in adults aged 60 years and older. Older adults are particularly vulnerable to chronic depression due to factors such as bereavement, social isolation, somatic diseases, and cognitive decline [4]. With increasing life expectancy, LLD has become a significant concern, negatively affecting quality of life [5]. Beyond its core symptomatology, LLD is linked to an increased risk of cardiovascular and cerebrovascular diseases, metabolic disorders, and a higher likelihood of developing dementia [6].

Clinical trials for acute treatment of depression in the elderly have demonstrated that even among patients who fall into similar diagnostic categories and receive the same treatment, the response to treatment can vary substantially. The somatic conditions contributing to LLD are not fully understood and likely involve interactions between various biological processes [7]. Potential factors include co-occurring anxiety symptoms, a history of recurrent depression, or early-stage neurodegenerative diseases such as Alzheimer’s disease (AD) [8, 9]. In the latter case, poor response may reflect a different underlying disorder rather than treatment inefficacy. This possibility has led to a growing interest in biomarkers associated with AD (amyloid and phosphorylated tau) and biomarkers for nonspecific processes involved in AD pathophysiology (axonal damage and astrogliosis), as tools to clarify whether neurodegenerative changes contribute to treatment resistance in LLD.

At the same time, aging-related biological pathways beyond AD have also been implicated in LLD. Studies involving patients with LLD and age-matched controls without depression have highlighted a connection between LLD and an aging-related molecular pattern, which was also associated with clinical and structural brain aging phenotypes [10]. Specifically, LLD patients showed increased plasma levels of senescence-associated secretory phenotype (SASP) proteins, which correlated with higher somatic comorbidity and poorer cognitive function. The release of SASP proteins, resulting from cellular senescence, is a key aging process. This accumulation of senescent cells in the brain is thought to create an inflammatory environment that may promote neurodegenerative diseases [11]. These processes could in turn exacerbate depressive symptoms and undermine response to psychotherapy.

Taken together, assessing AD-related biomarkers alongside SASP proteins could provide a framework for exploring distinct yet potentially interacting biological mechanisms underlying treatment heterogeneity in LLD. To date, however, no studies have examined their relevance for psychotherapy outcomes. In the present study, we sought to identify blood-based biomarkers that could shed light on the biological pathways underlying LLD and inform variability in response to psychotherapy. We analyzed data from 228 participants from the Cognitive Behavioral Therapy for Late-Life Depression (CBTlate) trial. For participants with available plasma samples, we examined the relationship between depressive symptom trajectories and levels of core plasma biomarkers associated with AD, biomarkers related to nonspecific processes involved in AD pathophysiology, and SASP proteins.

## Materials and methods

### Participants

Participants for the CBTlate trial were recruited from seven clinical research sites in Germany between October 2018 and November 2020. Inclusion criteria required participants to be at least 60 years old and meet the diagnostic criteria for moderate to severe MDD, as assessed by the Mini-International Neuropsychiatric Interview [12]. A total of 251 participants were randomized to receive 8 weeks of either specific cognitive behavioral therapy for LLD (LLD-CBT) or a nonspecific supportive intervention (SUI). Of these, 229 individuals were included in the intention-to-treat analysis. Clinical outcomes were assessed at baseline, week 5, post-treatment (week 10), and follow-up (month 6), using measures such as the 30-item Geriatric Depression Scale (GDS) [13], the 20-item Geriatric Anxiety Inventory (GAI) [14], and the Insomnia Severity Index (ISI) [15]. Cognitive status was assessed with the Mini-Mental State Examination (MMSE) and the Consortium to Establish a Registry for Alzheimer’s Disease (CERAD) neuropsychological test battery [16, 17]. Mild cognitive impairment (MCI) was algorithmically defined using base rate MCI criteria, which considers the number of deviant (>1 standard deviation) CERAD subtests after demographic adjustments [18, 19]. Blood samples were collected from participants at five centers (baseline, *N* = 101; follow-up, *N* = 73) who agreed to provide them. Since both treatments equally reduced depressive symptoms, participants were pooled for the subsequent analyses, regardless of treatment allocation. Detailed inclusion/exclusion criteria, randomization procedures, and treatment specifics are available in the published protocol [20]. All participants provided informed consent, and the study was approved by the local ethical committees (Institutional Review Board/Independent Ethics Committee) at each site. The trial is registered at ClinicalTrials.gov (NCT03735576) and DRKS (DRKS00013769).

### Blood collection

Blood was collected in EDTA tubes by antecubital venepuncture at the local sites. Plasma was separated by centrifugation and aliquoted and stored at −80 °C until further use. All aliquots were shipped to the central biorepository in Cologne for long-term storage.

### APOE genotyping

Leukocyte DNA was isolated from baseline EDTA-blood with Qiagen blood isolation kit according to the manufacturer’s instructions (Qiagen, Germany). *APOE* was genotyped using TaqMan technology, assay IDs 3084793_20 (SNP rs429358) and 904973_10 (SNP rs7412).

### AD biomarkers and biomarkers related to nonspecific processes involved in AD pathophysiology

Plasma levels of AD core biomarkers Aβ40, Aβ42, and P-tau181, and plasma levels of neurofilament light chain (NfL) and glial fibrillary acidic protein (GFAP), both biomarkers of nonspecific processes involved in AD pathophysiology (axonal damage and astrogliosis, respectively), were measured at baseline in all participants with a plasma sample available (*N* = 101). Aβ40, Aβ42, NfL, and GFAP were measured using the Neurology 4-plex E Simoa assay kit, and P-tau181 with the pTau181 Advantage V2 Simoa assay kit. Samples with a coefficient of variance exceeding 20% were re-measured, and if the second measurement also showed high variance, they were excluded from the analysis. Both assays were run using a Simoa HD-X platform at the DZNE Bonn.

### SASP measurement

The plasma levels of SASP proteins [21] were measured using Olink® Explore 3072 multiplex assay from Olink Proteomics in all participants with plasma samples at both baseline and follow-up (baseline, *N* = 73; follow-up, *N* = 73). Quality control (QC) procedures are described in the Supplementary Material. After QC completion, 44/58 SASP proteins available in this multiplex assay from all samples were included in the analysis. Protein levels are shown as normalized (log2-transformed) protein expression.

### Statistical analysis

All statistical analyses were conducted using R version 4.4.3. Multiple testing was corrected for false discovery rate (FDR) at an FDR-adjusted *p*-value (i.e., *q*-value) threshold of 0.05.

### Trajectory clustering

Single trajectories of GDS scores were clustered using the unsupervised machine learning method *k*-means for longitudinal data (kml) [22]. This method is particularly effective in situations with a low number of repeated measurements [23]. The model utilized all available data, including those from participants with incomplete follow-up. One participant had the follow-up assessment 417 days after the study baseline and was therefore excluded from subsequent analyses. Missing data were assumed to be missing at random and were imputed using the Copy Mean method [24]. Clustering quality was assessed using the nonparametric Calinski–Harabasz criterion, while Euclidean distance with Gower adjustment [25] was employed to determine the distance between trajectories. The analysis was conducted with the R package *kml.*

### Association between GDS clusters and Alzheimer’s plasma biomarkers or SASP proteins

Linear regression was used to evaluate the association, where the GDS cluster was the predictor of interest and the baseline plasma protein level was the outcome measure. Age at baseline, gender, and type of treatment were included as covariates. To assess whether MCI status moderated the relationship between protein levels and GDS cluster membership, logistic regression was performed using GDS cluster as outcome, and protein level, MCI status, age at baseline, gender, type of treatment, and the interaction term between protein level and MCI as covariates. To allow comparison, protein levels were *z*-transformed.

### Plasma CXCL5 dynamics

The trajectory of plasma CXCL5 level in each GDS cluster was assessed with linear mixed-effects models for repeated measures fit by maximum likelihood using the R package *lme4.* The models included all CXCL5 data available at baseline and follow-up assessment. Longitudinal plasma level was used as the outcome measure, and the fixed effects included time, GDS cluster, baseline age, and gender, and the interaction terms between time and all other covariates. Time was treated as a continuous variable. Since only two time points were available, a random intercept was included for each subject, but it was not possible to add a random slope of time for each participant. *t*-tests were calculated with Satterthwaite’s method using the R package *lmerTest.*

### Prediction of GDS clusters assignment

Three nested multivariate logistic models were fitted to predict GDS cluster assignment. All models had GDS clusters as the outcome measure. The first model was fitted, including baseline age, gender, first major depressive episode (MDE) before the age of 60 years, baseline MCI status, and baseline GDS, GAI, and ISI scores as predictors. In the second model, the baseline level of NfL was also included as a predictor. The last model was fitted using all the predictors from the second model, plus the baseline level of CXCL5. The area under the receiver-operator characteristic curve (AUC) for each model was calculated using the R package *pROC.* To allow comparison, all continuous variables were *z*-transformed.

## Results

### Participants characteristics at baseline

In this longitudinal study, we included 228 individuals diagnosed with LLD who participated in the CBTlate trial. Analysis of participant demographics at baseline revealed a predominance of females, constituting 65.8% of the cohort, with a mean age of 70.2 years. Additionally, participants had received a mean of 14.8 years of education. The majority of participants reported being in a relationship (60.5%) and not living alone (55.7%). Notably, 75.9% had experienced their first MDE before the age of 60 years, with 67.1% having received psychotherapy and 68.2% having utilized antidepressants throughout their lifetime. Assessments indicated a mean 30-item GDS score of 20.7, accompanied by a mean GAI score of 11.5 and a mean ISI score of 13.7. Cognitive assessments revealed a mean MMSE score of 29.1, with 15.8% of participants diagnosed with MCI (Supplementary Table 1).

### Participants can be clustered into two subgroups based on GDS trajectories

**Figure 1.**
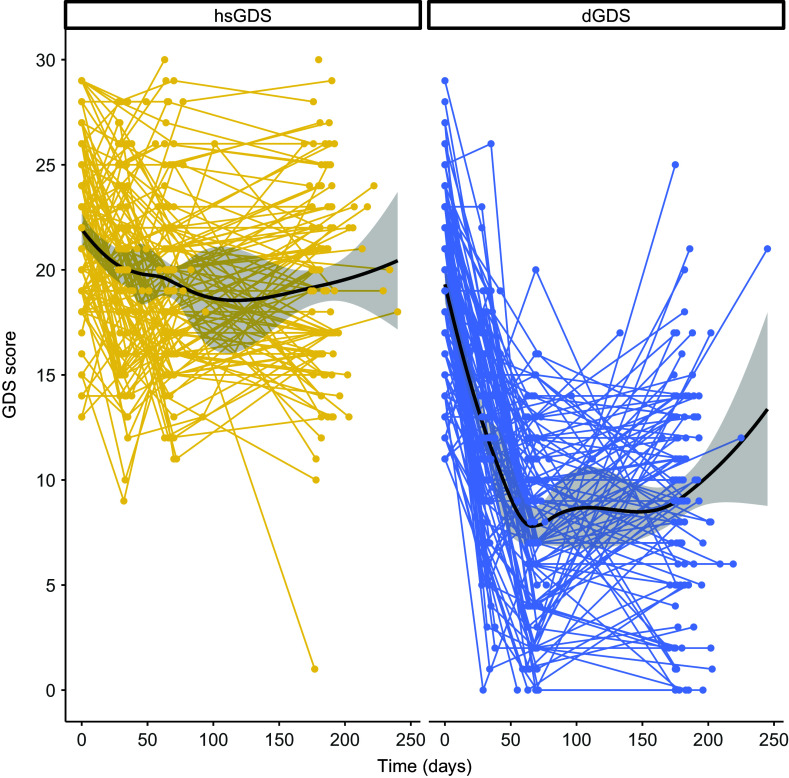
Clustering of GDS score trajectories over the course of treatment and follow-up. Spaghetti plots depict the trajectory of GDS scores within each cluster for every participant in the CBTlate trial (hsGDS, *N* = 119; dGDS, *N* = 110). Loess curves to smooth the trajectories were fit only for visualization purposes. GDS, Geriatric Depression Scale; hsGDS, high stable GDS cluster; dGDS, decreasing GDS cluster.

To identify subgroups of LLD patients with differing responses to psychotherapeutic treatment, we applied longitudinal *k*-means clustering to GDS score trajectories in all participants from the CBTlate trial. This approach allowed us to effectively model the evolution of depressive symptoms throughout the entire study period, rather than relying on cutoff points or defining remission or response based on a specific time point. The Calinski–Harabasz index suggested that the optimal number of clusters was two (Supplementary Figure 1). One cluster, including 51.8% of the participants, exhibited high and stable GDS scores throughout treatment (labeled as “high stable GDS” or “hsGDS”), while the remaining 48.2% comprised a second cluster demonstrating decreasing GDS scores (referred to as “decreasing GDS” or “dGDS”). In the latter, the majority exhibited decreasing GDS scores until the end of treatment, followed by either stabilization or an increase in GDS scores from the end of treatment until the follow-up assessment (Figure 1). hsGDS patients were significantly more likely to have an MDE history before the age of 60 years (*p* = 0.01) and to be diagnosed with MCI (*p* = 0.01). Additionally, they showed higher baseline GDS (*p* = 2.88 × 10^−6^), GAI (*p* = 4.39 × 10^−4^), and ISI (*p* = 1.09 × 10^−3^) scores. Although hsGDS participants tended to be slightly older, this difference was not statistically significant (*p* = 0.07) (Table 1).

**Table 1.** Demographic and clinical characteristics at baseline of participants with late-life depression from each GDS cluster

Characteristics	hsGDS (*N* = 118)	dGDS (*N* = 110)	***t*-test/*χ***^**2**^**-test**	*p*-value
Gender (female, %)	66.1	65.5	*χ*^2^(1) = 0	1
Age (mean [SD])	71 (7)	69.3 (7)	*t*(225.49) = 1.85	0.07
BMI (mean [SD])	26 (5)	26.4 (5)	*t*(224.51) = −0.5	0.62
Education (years, mean [SD])	15 (3)	14.5 (3)	*t*(222.7) = 1.33	0.18
In a relationship (%)	61.9	59.1	*χ*^2^(1) = 0.09	0.77
Living alone (%)	41.5	47.3	*χ*^2^(1) = 0.48	0.49
Age at first MDE (mean [SD])	41 (20)	44.7 (21)	*t*(214.92) = −1.35	0.18
First MDE before 60 years of age (%)	83.2	68.2	*χ*^2^(1) = 5.93	0.01
Number of MDE (mean [SD])	4 (7)	4.8 (12)	*t*(167.77) = −0.6	0.55
Duration of the current MDE (months, mean [SD])	41.4 (95)	31.6 (55)	*t*(177.93) = 0.94	0.35
Psychiatric treatment				
Inpatient (lifetime, %)	44.1	34.5	*χ*^2^(1) = 1.78	0.18
Outpatient (lifetime, %)	16.1	21.8	*χ*^2^(1) = 0.87	0.35
Outpatient psychotherapy (lifetime, %)	72	61.8	*χ*^2^(1) = 2.25	0.13
Antidepressants				
Lifetime (%)	69.7	66.7	*χ*^2^(1) = 0.11	0.74
Current use (%)	39.8	44.5	*χ*^2^(1) = 0.34	0.56
Suicidal tendency (%)	10.2	9.1	*χ*^2^(1) = 0	0.96
GDS (mean [SD])	21.9 (4)	19.3 (4)	*t*(219.46) = 4.8	2.89 × 10^−6^
GAI (mean [SD])	12.5 (4)	10.5 (4)	*t*(220.08) = 3.57	4.39 × 10^−4^
MMSE (mean [SD])	29 (1)	29.1 (1)	*t*(225.7) = −0.53	0.6
ISI (mean [SD])	15 (6)	12.4 (6)	*t*(218.04) = 3.31	1.09 × 10^−3^
MCI (%)	22	9.1	*χ*^2^(1) = 6.23	0.01
Treatment (LLD-CBT, %)	50	50	*χ*^2^(1) = 0	1

Abbreviations: hsGDS, high stable GDS; dGDS, decreasing GDS; GDS, Geriatric Depression Scale; SD, standard deviation; MDE, major depressive episode; GAI, Geriatric Anxiety Inventory; MMSE, Mini-Mental State Examination; ISI, Insomnia Severity Index; MCI, mild cognitive impairment; LLD-CBT; late-life depression-specific cognitive behavioral therapy; *p*-value, nominal *p*-value.

### Plasma level of NfL is increased in hsGDS participants

**Figure 2.**
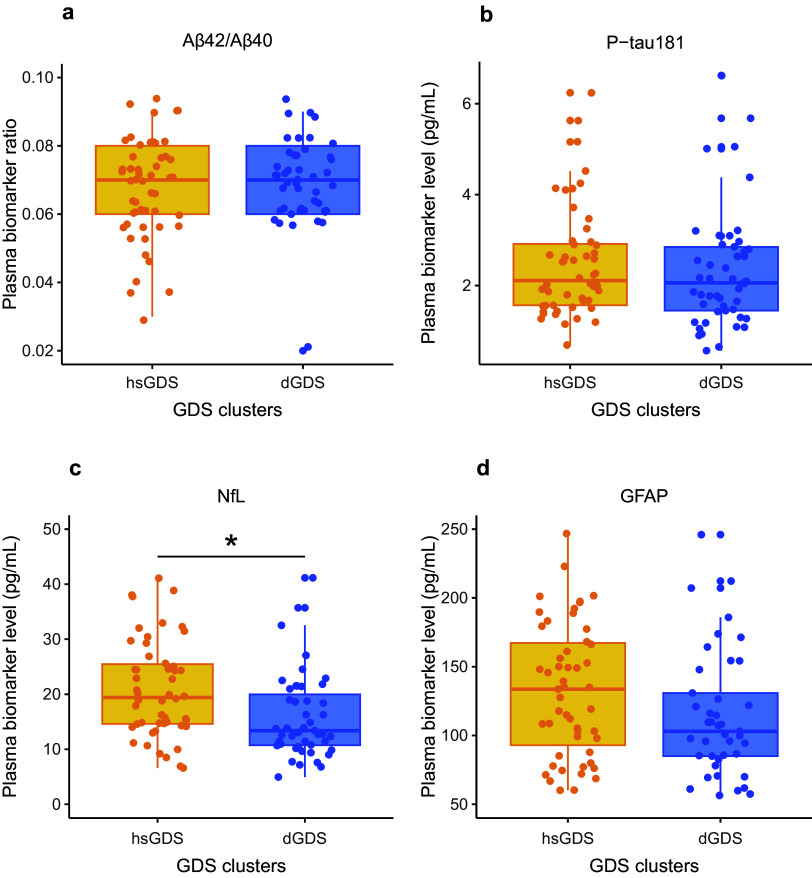
Association between Alzheimer’s disease plasma biomarkers and GDS clusters. Box plots indicate ratios/levels of core Alzheimer’s disease plasma biomarkers and biomarkers of nonspecific processes involved in Alzheimer’s disease pathophysiology in hsGDS (*N* = 52) and dGDS clusters (*N* = 49). GDS, Geriatric Depression Scale; hsGDS, high stable GDS cluster; dGDS, decreasing GDS cluster. *p < 0.05.

Next, we evaluated whether the differences observed between the hsGDS and dGDS clusters could be attributed to underlying organic brain damage or a neurodegenerative process. Herein, we quantified AD core plasma biomarkers and biomarkers of nonspecific processes indicating potential organic brain damage. Specifically, we evaluated plasma Aβ42/Aβ40 ratio and plasma level of P-tau181 (AD-specific), and plasma levels of NfL and GFAP (nonspecific brain damage) in participants with available samples at baseline (hsGDS, *N* = 52; dGDS, *N* = 49). The demographic and clinical characteristics of this subset of participants are detailed in Supplementary Table 2. Notably, no significant differences were observed between clusters in the biomarkers specific for AD (Aβ42/Aβ40 ratio: *β* = −0.16, SE = 0.22, *p* = 0.47; P-tau181: *β* = −0.04, SE = 0.20, *p* = 0.84). GFAP, a biomarker for astrogliosis, exhibited a trend toward increased levels in the hsGDS cluster, but this difference did not achieve statistical significance (*β* = 0.28, SE = 0.19, *p* = 0.15). However, we did find a significantly higher NfL level, a biomarker for axonal damage, in the hsGDS cluster (*β* = 0.40, SE = 0.18, *p* = 2.51 × 10^−2^), suggesting that hsGDS patients may exhibit underlying brain damage (Figure 2). Because plasma NfL is often elevated in individuals with MCI, we next examined whether the observed cluster differences in NfL could be explained by MCI status. To this end, we tested the interaction between NfL levels and MCI diagnosis in predicting cluster membership. The interaction was not significant (*β* = 0.34, SE = 0.63, *p* = 0.59), suggesting that higher NfL levels in the hsGDS cluster are not merely a reflection of concurrent MCI.

### Plasma level of CXCL5 is decreased in hsGDS participants

**Figure 3.**
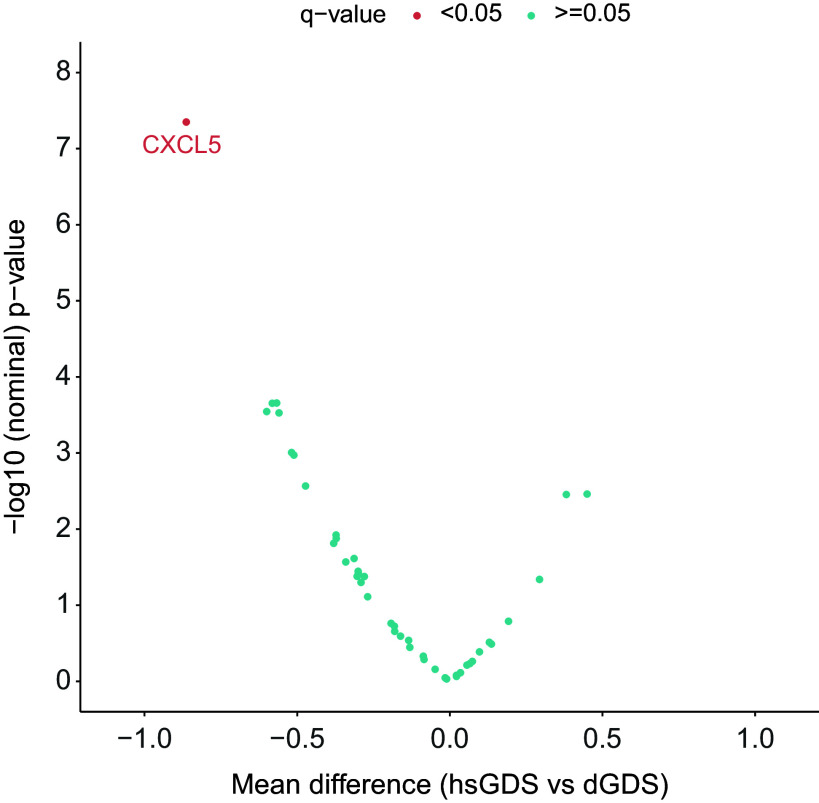
Association between SASP protein levels and GDS clusters. The volcano plot indicates the mean difference in protein level between hsGDS (*N* = 32) and dGDS (*N* = 41) clusters. The dGDS cluster was used as the reference category. Protein levels were *z*-transformed to allow comparison. *q*-value, false discovery rate-corrected *p*-value; GDS, Geriatric Depression Scale; hsGDS, high stable GDS cluster; dGDS, decreasing GDS cluster.

To gain further insight into the potential pathophysiological pathways driving the depressive symptom trajectories in both GDS clusters, we explored several SASP proteins known to correlate with increased medical comorbidities and poorer cognitive function in LLD patients [10]. We assessed the levels of 44 SASP proteins, both at baseline and follow-up, in all participants with matched plasma samples at both time points (hsGDS, *N* = 32; dGDS, *N* = 41). Detailed demographic and clinical characteristics of this subset of participants are provided in Supplementary Table 3. At baseline, we identified a significantly lower concentration of six plasma proteins in the hsGDS cluster. However, correction for multiple testing revealed that only plasma CXCL5 remained significantly lower in the hsGDS cluster (*β* = −0.86, SE = 0.24, *q* = 0.03) (Figure 3, Supplementary Figure 2, and Supplementary Table 4). Since SASP proteins have been associated with cognitive function, we also examined whether MCI diagnosis moderated the association between CXCL5 levels and cluster membership. The interaction was not significant (*β* = −0.42, SE = 1.02, *p* = 0.68), consistent with our findings for NfL, indicating that lower CXCL5 levels in the hsGDS cluster were not accounted for by MCI status. Next, we investigated whether plasma CXCL5 levels changed during the course of treatment. We found no significant association between CXCL5 level and time (*β* = 0.31, SE = 0.19, *p* = 0.11), and no effect of GDS clusters on the longitudinal CXCL5 level (*β* = 0.06, SE = 0.04, *p* = 0.12) (Supplementary Figure 3 and Supplementary Table 5).

### Plasma level of CXCL5 improves the discrimination of GDS clusters beyond clinical data

**Figure 4.**
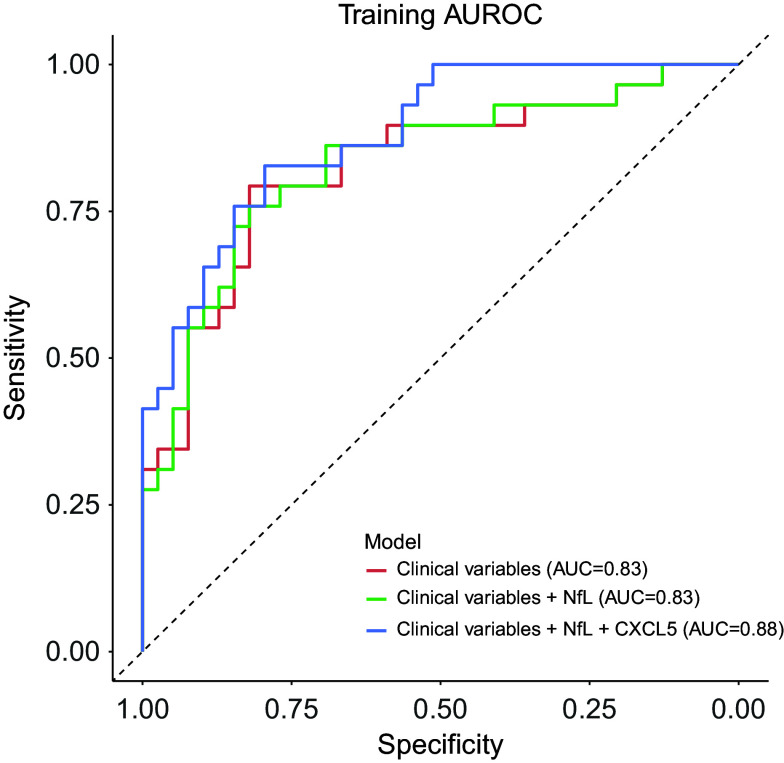
Discrimination performance of clinical variables and plasma levels of NfL and CXCL5 to predict GDS clusters assignment. The plot indicates the area under the receiver-operator characteristic curve (AUC) for each predictive model. Clinical variables included the first depressive episode before the age of 60 years, MCI status, GDS, GAI, and ISI scores, as well as age and gender. MCI, mild cognitive impairment; GDS, Geriatric Depression Scale; GAI, Geriatric Anxiety Inventory; ISI, Insomnia Severity Index.

Given the differential concentrations of plasma NfL and CXCL5 levels in the two GDS clusters, we sought to evaluate their potential utility in improving the identification of participants whose GDS scores may change during treatment, beyond demographic and clinical characteristics. To achieve this, we applied three nested logistic models with dGDS group as outcome and compared their discrimination capacity. The first model (base model) included age, gender, first MDE before the age of 60 years, MCI status, as well as GDS, GAI, and ISI scores as predictors. This base model exhibited an AUC of 0.83, with only age, gender, and first MDE before the age of 60 years showing significant associations with the outcome, conditional on the rest of the predictors. The second model incorporated the plasma level of NfL into the base model. However, only the first MDE before the age of 60 years remained significantly associated with the outcome. The addition of NfL yielded an AUC of 0.85, but did not improve the goodness-of-fit (*χ*^2^(1) = 2.90, *p* = 0.08) compared to the base model. Finally, the third model integrated all the predictors from the second model with the plasma level of CXCL5. This model achieved an AUC of 0.88, with only age, first MDE before the age of 60 years, and CXCL5 level demonstrating significant associations with the outcome. Compared to both base and second models, the third model showed superior goodness-of-fit (versus the base model: *χ*^2^(2) = 11.21, *p* = 3.69 × 10^−3^; versus the second model: *χ*^2^(1) = 8.31, *p* = 3.94 × 10^−3^), indicating that plasma CXCL5 level may enhance the discrimination between clusters beyond demographic and clinical data and plasma NfL level (Figure 4 and Supplementary Table 6).

## Discussion

With the increasing elderly population, the prevalence of LLD is rising, leading to significant impacts on quality of life and other medical outcomes. However, treatment for LLD remains challenging due to the unclear biological mechanisms underlying the condition [7]. In this longitudinal study, we aimed to identify biomarkers predictive of psychotherapeutic treatment response to better understand the pathophysiological processes in LLD. Our analysis revealed two distinct treatment response groups: one with high and stable GDS scores throughout treatment (hsGDS, which could be referred to as nonresponders) and another with decreasing GDS scores during treatment, followed by stabilization or an increase at follow-up (dGDS, responders). We found that nonresponders exhibited a higher baseline plasma NfL level, indicative of axonal damage, and a lower plasma CXCL5 level, reflecting inflammation and cellular aging, suggesting that these biomarkers may serve to predict treatment outcomes in LLD.

The heterogeneity of LLD has been previously studied by modeling depression trajectories in observational studies [26] or by clustering patients into subtypes based on their depressive symptoms at baseline [27]. Leveraging the design of the CBTlate trial, we adopted a novel approach by clustering patients according to the evolution of their depressive symptoms throughout treatment and follow-up. This method provided a more dynamic understanding of patient responses, revealing the heterogeneity of the trajectories and allowing the identification of associated clinical and biological factors that predict treatment outcome and could eventually guide personalized interventions. We observed that at baseline, nonresponders exhibited a higher frequency of a first MDE before the age of 60 years, a diagnosis of MCI, and more severe depression, anxiety, and sleep disorders. These results align with previous findings indicating that older adults with high severity of depression and anxiety, sleep disturbance, and cognitive impairment show worse treatment response [27].

Although LLD has been associated with an increased risk of dementia, including vascular dementia and dementia of the Alzheimer’s type (DAT) [28], their relationship remains complex and inconclusive [29]. While some studies suggest depression as a risk factor for AD, others propose depression as an early manifestation of AD. However, most evidence comes from epidemiological data, and only a limited number of studies have assessed AD biomarkers in LLD [30]. To improve understanding of the underlying mechanisms of LLD and to identify biological determinants of psychotherapeutic treatment response, we compared responders and nonresponders in plasma levels of AD core biomarkers (Aβ42/Aβ40 ratio, P-tau181), in GFAP (a marker of astrogliosis), and in NfL (a marker of axonal damage). Even in the absence of a control group without LLD or a group with confirmed AD, such comparisons can reveal whether biological processes commonly implicated in AD are differentially expressed in relation to psychotherapy outcomes in LLD and may highlight mechanistic overlaps with neurodegenerative disorders.

Plasma Aβ42/Aβ40 ratio, P-tau181, and GFAP can show alterations years before DAT onset, whereas NfL is typically altered only at later stages, when cognitive impairment becomes apparent [31]. In our study, we observed no significant differences in Aβ42/Aβ40 ratio, P-tau181, or GFAP. These results are in line with a large meta-analysis on AD plasma biomarkers in LLD, which showed no association between depressive symptoms and these plasma biomarkers [30]. In contrast, nonresponders exhibited higher baseline plasma levels of NfL. If nonresponders in our cohort were at an AD stage where NfL changes were detectable, we would also expect to see differences in Aβ42/Aβ40 and P-tau181. The absence of such differences suggests that AD pathology detectable through these biomarkers is unlikely to explain the distinct treatment trajectories observed here, though in our setting, their involvement in LLD overall cannot be excluded.

In the general older population without dementia, higher plasma NfL levels are associated with cognitive decline and a larger burden of primarily white matter pathology [32]. However, the role of NfL in LLD remains unclear [33]. Elevated plasma NfL levels have previously been reported in middle-aged individuals with MDD compared to controls [34, 35], and higher levels were associated with more severe depressive symptoms in LLD patients [35]. Our study is the first to link elevated plasma NfL levels and nonresponse to psychotherapy, supporting the view that treatment-resistant MDD may be linked to persistent axonal damage that disrupts communication within mood-regulating neural circuits [36]. Furthermore, emerging evidence points to the involvement of myelin pathologies in MDD [37–39]. Disruptions in axon–myelin interactions can impair conduction velocity and destabilize the axonal cytoskeleton, thereby contributing to axonal damage. In line with this interpretation, we observed that nonresponders were more likely to have a diagnosis of MCI. This finding is consistent with large longitudinal studies showing that individuals with high or persistent depressive symptom trajectories perform worse on cognitive tasks and experience greater cognitive decline compared with those with low or improving symptom trajectories [40–42]. Although nonresponders had both higher NfL levels and a higher prevalence of MCI, the association between NfL and cluster membership was not dependent on MCI status. These results suggest that elevated NfL may reflect axonal damage and cognitive deficits associated with treatment resistance in LLD, even in participants who do not meet criteria for MCI.

We also explored the relationship between LLD and age-related molecular patterns, particularly SASP dysregulation. Though we compared the plasma levels of several SASP proteins, only CXCL5 level was significantly lower in nonresponders. Plasma level of CXCL5 improved the discrimination between GDS clusters beyond demographic and clinical data, suggesting its potential as a biomarker for predicting psychotherapeutic treatment response. CXCL5, also known as epithelial-derived neutrophil-activating peptide 78 (ENA-78), is an inflammatory chemokine produced alongside interleukin (IL)-8 in response to stimulation by IL-1 or tumor necrosis factor-α (TNF-α). Chemokines are chemotactic cytokines, and previous research has shown that they are involved in neuron–glia communication and synaptic transmission [43]. Our findings align with a prior clinical trial in adults with MDD, which reported that nonresponders to an 8-week venlafaxine treatment had a lower baseline plasma level of CXCL5 compared to responders [44]. Reduced peripheral CXCL5 levels in MDD have been proposed to reflect elevated CXCL5 activity in the brain, possibly due to activation of the hypothalamic–pituitary–adrenal (HPA) system by negative feedback [44]. In this context, upregulated CXCL5 in the brain may contribute to neuroinflammation. However, we did not observe significant changes over time in the plasma level of CXCL5 in any GDS cluster. This stability across the study period suggests that plasma CXCL5 may represent a trait-like characteristic rather than a marker that can be modified by the psychotherapeutic treatment used in the CBTlate study.

Interestingly, increased NfL levels in MDD have been associated with increased levels of proinflammatory cytokines, particularly TNF-α [34], a key stimulator of CXCL5 production. An increased level of TNF-α plays a significant role in MDD [45] and the HPA system [46]. Given this relationship, one plausible model is that axonal damage, reflected in elevated NfL, could promote neuroinflammation and thereby increase TNF-α signaling. TNF-α, in turn, may drive CXCL5 production within the brain, while lower CXCL5 is observed peripherally. This mechanism would be compatible with nonresponse arising from underlying axonal pathology and inflammatory dysregulation. Such processes could reflect either a limited capacity to repair damaged axons, a reduced regenerative response to treatment, or early manifestations of a neurodegenerative disorder. While our current data do not allow us to disentangle these possibilities, our findings support the broader interpretation that elevated plasma NfL together with decreased plasma CXCL5 reflect an interplay between aging, inflammation, and axonal damage that may characterize a specific LLD subtype with reduced treatment responsiveness.

Our study has several limitations. First, the absence of a control group without depression or a waiting-list group limits our ability to assess NfL and CXCL5 levels in healthy or untreated individuals. Second, due to the lack of cerebrospinal fluid samples or magnetic resonance imaging data, we cannot confirm the presence of axonal damage in nonresponders. Third, our study focused on two types of psychotherapy, limiting the generalizability of our findings to other forms of treatment. Finally, while plasma CXCL5 showed promising potential as a biomarker for treatment response, further validation in independent, larger cohorts is necessary.

In conclusion, our study highlights the heterogeneity of LLD treatment response, identifying two distinct trajectories associated with baseline levels of NfL and CXCL5. Nonresponders showed higher NfL and lower CXCL5 levels, suggesting a complex interplay between neuroinflammation, axonal damage, and aging that may hinder treatment response. Plasma CXCL5, in particular, holds potential as a biomarker for predicting treatment outcomes, paving the way for more personalized treatment approaches in LLD.

## Supporting information

10.1192/j.eurpsy.2026.10153.sm001 Martino-Adami et al. supplementary material Martino-Adami et al. supplementary material

## Data Availability

The data that support the findings of this study are available from the corresponding authors upon reasonable request.

## Supplementary material

The supplementary material for this article can be found at http://doi.org/10.1192/j.eurpsy.2026.10153.
