# Supplementary material for: Exploring blood-based biomarkers in late-life depression: Correlates of psychotherapeutic treatment outcomes
Source: Eur Psychiatry. 2026 Jan 23;69(1):e18. doi: 10.1192/j.eurpsy.2026.10153 (PMC12925672; doi:10.1192/j.eurpsy.2026.10153)
Supplement: Martino-Adami et al. supplementary material [file S0924933826101539sup001.docx]

**Supplementary material**

**Quality control of SASP measurements**

Quality control (QC) of Olink® Explore 3072 multiplex assay from Olink Proteomics was conducted on (i) each specific combination of sample and protein, (ii) each sample, and (iii) each protein. For each specific combination of sample and protein to pass the QC, (a) the sample should have at least 500 reads, (b) the sample deviation from the median value of the internal amplification control should not exceed +/- 0.3 NPX, (c) the protein should have a deviation of the median value of the internal negative controls lower or equal than 5 standard deviations, and (d) the protein value should be equal or higher than the limit of detection. All specific combinations of sample and protein not meeting these criteria were excluded. Then, samples with excluded values in at least 80% of all proteins measured and proteins with more than 10% of excluded values were eliminated.

**
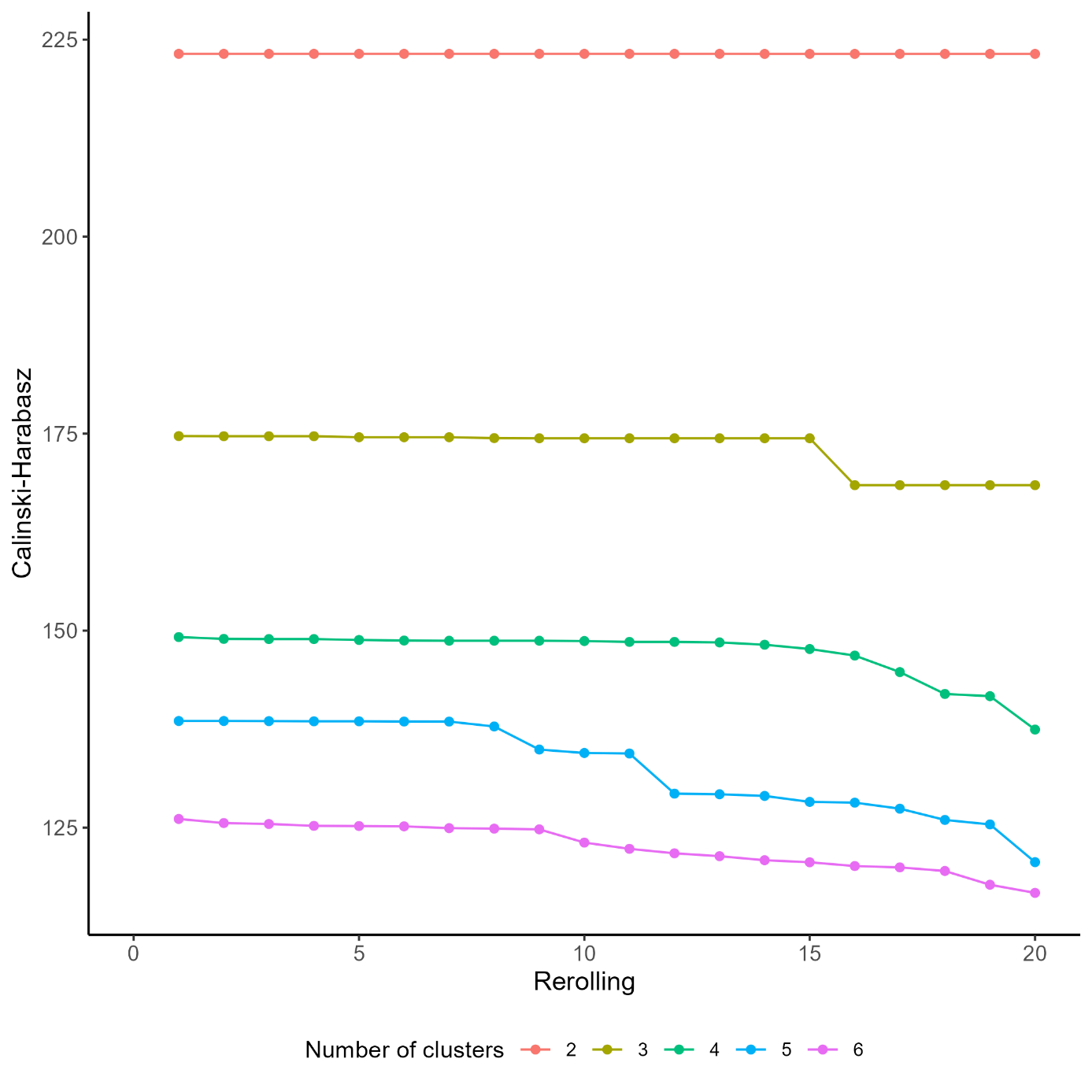
**

**Supplementary Figure 1. Evaluation of the optimal number of clusters for GDS trajectories.** Calinski-Harabasz criterion was used to evaluate the optimal number of clusters, i.e., the solution with the largest between-cluster variance and smallest within-cluster variance (highest Calinski-Harabasz index value).

**
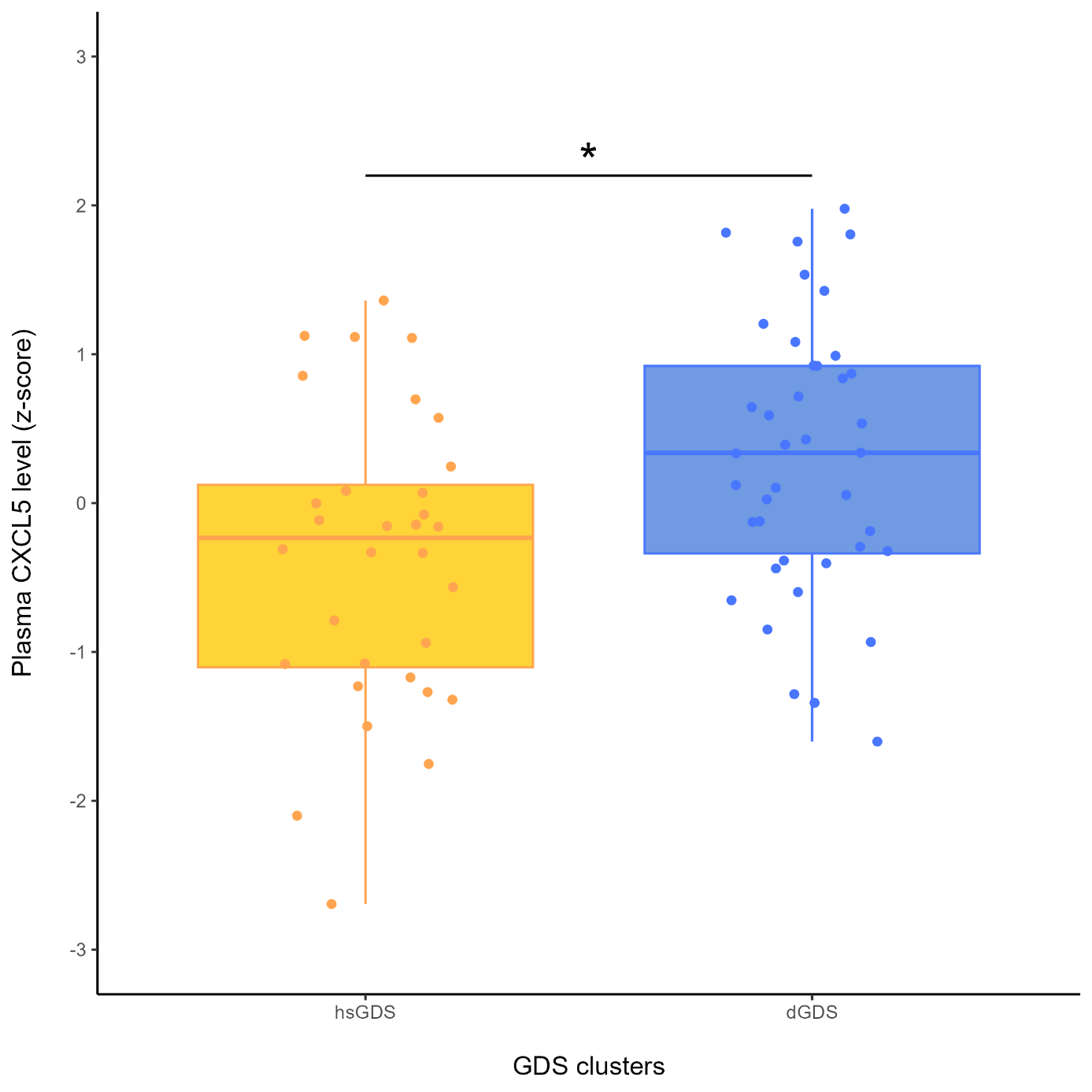
**

**Supplementary Figure 2. Plasma level of CXCL5 in each GDS cluster.** Boxes represent the *z*-transformed plasma level of CXCL5 in each GDS cluster. Comparison was performed adjusting for age at baseline, gender, and type of treatment during the trial. hsGDS, high and stable GDS; dGDS, decreasing GDS; GDS, geriatric depression scale. **q* (false discovery rate-adjusted *p*-value)<0.05.

**
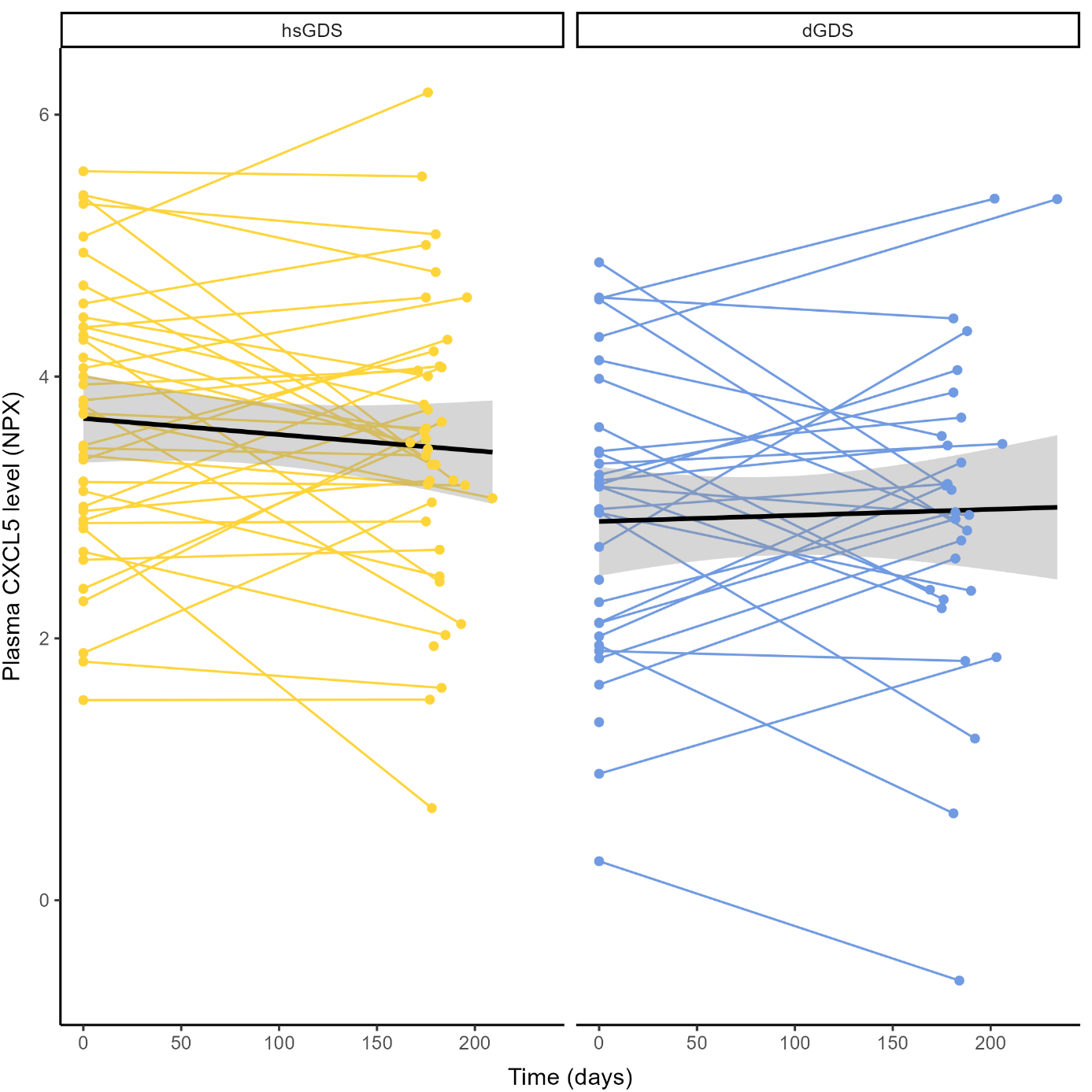
**

**Supplementary Figure 3. Longitudinal plasma level of CXCL5 in GDS clusters.** Spaghetti plots show the change in CXCL5 level from baseline until follow-up assessment in hsGDS (N=32) and dGDS clusters (N=41). Each circle represents a protein measurement. Regression lines were fitted for each group only for visualisation purposes. GDS, geriatric depression scale; hsGDS, high stable GDS cluster; dGDS, decreasing GDS cluster, NPX, normalised protein expression.

**Supplementary Table 1. Demographic and clinical characteristics at baseline of participants with late-life depression.**

| **Characteristics** | **All participants**  **(N=228)** |
| --- | --- |
| Gender (female, %) | 65.8 |
| Age (mean (SD)) | 70.2 (7) |
| BMI (mean (SD)) | 26.2 (5) |
| Education (years, mean (SD)) | 14.8 (3) |
| In a relationship (%) | 60.5 |
| Living alone (%) | 44.3 |
| Age at first MDE (mean (SD)) | 42.8 (20) |
| First MDE before 60 years of age (%) | 75.9 |
| Number of MDE (mean (SD)) | 4.4 (10) |
| Duration of the current MDE (months, mean (SD)) | 36.7 (78) |
| Psychiatric treatment |  |
| Inpatient (lifetime, %) | 39.5 |
| Outpatient (lifetime, %) | 18.9 |
| Outpatient psychotherapy (lifetime, %) | 67.1 |
| Antidepressants |  |
| Lifetime (%) | 68.2 |
| Current use (%) | 42.1 |
| Suicidal tendency (%) | 9.6 |
| GDS (mean (SD)) | 20.7 (4) |
| GAI (mean (SD)) | 11.5 (4) |
| MMSE (mean (SD)) | 29.1 (1) |
| ISI (mean (SD)) | 13.7 (6) |
| MCI (%) | 15.8 |
| Treatment (LLD-CBT, %) | 50 |

SD, standard deviation; MDE, major depressive episode; GDS, geriatric depression scale; GAI, geriatric anxiety inventory; MMSE, mini-mental state examination; ISI, insomnia severity index; MCI, mild cognitive impairment; LLD-CBT; late-life depression-specific cognitive behavioral therapy.

**Supplementary Table 2. Demographic and clinical characteristics at baseline of participants with late-life depression from each GDS cluster with Alzheimer’s disease biomarker data.**

| **Characteristics** | **All**  **(N=101)** | **hsGDS**  **(N=52)** | **dGDS**  **(N=49)** | ***t*-test/χ^2^-test** | ***p*-value** |
| --- | --- | --- | --- | --- | --- |
| Gender (female, %) | 62.4 | 54 | 71 | χ^2^(1) = 2.62 | 0.11 |
| Age (mean (SD)) | 70.9 (7) | 72.1 (7) | 69.5 (7) | *t*(98.99) = 1.87 | 0.06 |
| BMI (mean (SD)) | 26.5 (6) | 26.2 (6) | 26.7 (6) | *t*(98.99) = -0.42 | 0.67 |
| *APOE*-ε4 (%) | 30.7 | 23 | 39 | χ^2^(1) = 2.23 | 0.14 |
| Education (years, mean (SD)) | 15 (3) | 15.2 (3) | 14.8 (3) | *t*(98.93) = 0.76 | 0.45 |
| In a relationship (%) | 65.3 | 71 | 59 | χ^2^(1) = 1.11 | 0.29 |
| Living alone (%) | 39.6 | 35 | 45 | χ^2^(1) = 0.8 | 0.37 |
| Age at first MDE (mean (SD)) | 42.8 (21) | 43.2 (20) | 42.3 (21) | *t*(93.29) = 0.23 | 0.82 |
| First MDE before 60 years of age (%) | 76 | 82 | 70 | χ^2^(1) = 1.15 | 0.28 |
| Number of MDE (mean (SD)) | 4.5 (9) | 4.6 (9) | 4.3 (8) | *t*(88.52) = 0.16 | 0.87 |
| Duration of the current MDE (months, mean (SD)) | 34.7 (50) | 35 (45) | 34.4 (55) | *t*(88.9) = 0.06 | 0.95 |
| Psychiatric treatment |  |  |  |  |  |
| Inpatient (lifetime, %) | 32.7 | 37 | 29 | χ^2^(1) = 0.41 | 0.52 |
| Outpatient (lifetime, %) | 13.9 | 14 | 14 | χ^2^(1) = 0 | 1 |
| Outpatient psychotherapy (lifetime, %) | 69.3 | 69 | 69 | χ^2^(1) = 0 | 1 |
| Antidepressants |  |  |  |  |  |
| Lifetime (%) | 63.6 | 72 | 55 | χ^2^(1) = 2.37 | 0.12 |
| Current use (%) | 36.6 | 39 | 35 | χ^2^(1) = 0.03 | 0.85 |
| Suicidal tendency (%) | 12.9 | 12 | 14 | χ^2^(1) = 0.01 | 0.91 |
| GDS (mean (SD)) | 20.9 (4) | 21.7 (4) | 20.1 (4) | *t*(98.45) = 1.94 | 0.06 |
| GAI (mean (SD)) | 11.8 (4) | 12.6 (4) | 10.8 (5) | *t*(95.92) = 2.06 | 0.04 |
| MMSE (mean (SD)) | 29.1 (1) | 29 (1) | 29.2 (1) | *t*(98.74) = -0.9 | 0.37 |
| ISI (mean (SD)) | 13.9 (6) | 14.6 (6) | 13.3 (6) | *t*(98.99) = 1.04 | 0.30 |
| MCI (%) | 14.9 | 21 | 8 | χ^2^(1) = 2.42 | 0.12 |
| Treatment (LLD-CBT, %) | 47.5 | 46 | 22 | χ^2^(1) = 0.01 | 0.93 |

hsGDS, high and stable GDS; dGDS, decreasing GDS; GDS, geriatric depression scale; SD, standard deviation; MDE, major depressive episode; GAI, geriatric anxiety inventory; MMSE, mini-mental state examination; ISI, insomnia severity index; MCI, mild cognitive impairment; LLD-CBT; late-life depression-specific cognitive behavioral therapy; *P*-value, nominal *P*-value (hsDGS vs dGDS).

**Supplementary Table 3. Demographic and clinical characteristics at baseline of late-life depression participants from each GDS cluster with SASP data.**

| **Characteristics** | **All**  **(N=73)** | **hsGDS**  **(N=32)** | **dGDS**  **(N=41)** | ***t*-test/χ^2^-test** | ***p*-value** |
| --- | --- | --- | --- | --- | --- |
| Gender (female, %) | 68.5 | 56 | 78 | χ^2^(1) = 3.01 | 0.07 |
| Age (median (IQR)) | 70.4 (7) | 72.6 (8) | 68.6 (6) | *t*(61.41) = 2.4 | 0.02 |
| BMI (median (IQR)) | 26.6 (6) | 26.9 (7) | 26.4 (5) | *t*(52.82) = 0.32 | 0.75 |
| *APOE*-ε4 (%) | 30.1 | 19 | 39 | χ^2^(1) = 2.61 | 0.07 |
| Education (years, mean (SD)) | 14.8 (3) | 15.2 (3) | 14.4 (3) | *t*(62.33) = 1.06 | 0.29 |
| In a relationship (%) | 65.8 | 75 | 59 | χ^2^(1) = 1.49 | 0.3 |
| Living alone (%) | 41.1 | 34 | 46 | χ^2^(1) = 1.65 | 0.38 |
| Age at first MDE (mean (SD)) | 43 (21) | 41.7 (21) | 44 (21) | *t*(63.22) = -0.47 | 0.64 |
| First MDE before 60 years of age (%) | 75.7 | 87 | 68 | χ^2^(1) = 2.46 | 0.09 |
| Number of MDE (mean (SD)) | 4.4 (8) | 3.9 (8) | 4.7 (9) | *t*(58.01) = -0.4 | 0.69 |
| Duration of the current MDE (months, mean (SD)) | 37 (51) | 42.6 (53) | 32.6 (50) | *t*(62.62) = 0.81 | 0.42 |
| Psychiatric treatment |  |  |  |  |  |
| Inpatient (lifetime, %) | 31.5 | 38 | 27 | χ^2^(1) = 0.52 | 0.44 |
| Outpatient (lifetime, %) | 58.9 | 69 | 51 | χ^2^(1) = 0.37 | 0.15 |
| Outpatient psychotherapy (lifetime, %) | 65.8 | 66 | 66 | χ^2^(1) = 0 | 1 |
| Antidepressants |  |  |  |  |  |
| Lifetime (%) | 58.3 | 68 | 51 | χ^2^(1) = 1.36 | 0.23 |
| Current use (%) | 38.4 | 44 | 34 | χ^2^(1) = 0.35 | 0.48 |
| Suicidal tendency (%) | 15.1 | 16 | 15 | χ^2^(1) = 0 | 1 |
| GDS (mean (SD)) | 21.3 (4) | 22 (4) | 20.7 (4) | *t*(68.49) = 1.41 | 0.16 |
| GAI (mean (SD)) | 11.9 (4) | 12.7 (3) | 11.3 (5) | *t*(70.61) = 1.44 | 0.15 |
| MMSE (mean (SD)) | 29.1 (1) | 28.9 (1) | 29.2 (1) | *t*(54.58) = -1.48 | 0.14 |
| ISI (mean (SD)) | 13.8 (6) | 14.9 (6) | 12.9 (6) | *t*(67.48) = 1.43 | 0.16 |
| MCI (%) | 13.7 | 22 | 7 | χ^2^(1) = 2.11 | 0.1 |
| Treatment (LLD-CBT, %) | 43.8 | 38 | 49 | χ^2^(1) = 0.53 | 0.35 |

SASP, senescence-associated secretory phenotype; hsGDS, high and stable GDS; dGDS, decreasing GDS; GDS, geriatric depression scale; SD, standard deviation; MDE, major depressive episode; GAI, geriatric anxiety inventory; MMSE, mini-mental state examination; ISI, insomnia severity index; MCI, mild cognitive impairment; LLD-CBT; late-life depression-specific cognitive behavioral therapy; *P*-value, nominal *P*-value (hsGDS vs dGDS).

**Supplementary Table 4. Association between SASP protein levels and GDS clusters.**

| **Protein** | **β** | **SE** | ***p*-value** | ***q*-value** |
| --- | --- | --- | --- | --- |
| CXCL5 | -0.86 | 0.24 | 6.43x10^-4^ | 0.03 |
| CCL8 | -0.57 | 0.25 | 0.03 | 0.26 |
| SERPINE1 | -0.58 | 0.26 | 0.03 | 0.26 |
| CCL25 | -0.60 | 0.27 | 0.03 | 0.26 |
| CCL16 | -0.56 | 0.25 | 0.03 | 0.26 |
| SERPINE2 | -0.52 | 0.26 | 0.05 | 0.32 |
| EGF | -0.51 | 0.26 | 0.05 | 0.32 |
| VEGFA | -0.47 | 0.26 | 0.08 | 0.38 |
| IGFBP4 | 0.45 | 0.26 | 0.09 | 0.38 |
| IGFBP2 | 0.38 | 0.22 | 0.09 | 0.38 |
| AREG | -0.37 | 0.25 | 0.15 | 0.55 |
| CXCL3 | -0.37 | 0.26 | 0.15 | 0.55 |
| IL1B | -0.38 | 0.27 | 0.16 | 0.55 |
| EGFR | -0.31 | 0.24 | 0.20 | 0.57 |
| CCL13 | -0.34 | 0.27 | 0.21 | 0.57 |
| TNFRSF10C | -0.30 | 0.25 | 0.24 | 0.57 |
| MMP1 | -0.30 | 0.25 | 0.24 | 0.57 |
| CXCL1 | -0.30 | 0.26 | 0.25 | 0.57 |
| MMP3 | -0.28 | 0.24 | 0.25 | 0.57 |
| HRG | 0.29 | 0.26 | 0.26 | 0.57 |
| IGFBP3 | -0.29 | 0.26 | 0.27 | 0.57 |
| HGF | -0.27 | 0.27 | 0.33 | 0.66 |
| TIMP2 | 0.19 | 0.26 | 0.45 | 0.85 |
| CXCL11 | -0.19 | 0.26 | 0.47 | 0.85 |
| ICAM1 | -0.18 | 0.26 | 0.48 | 0.85 |
| CXCL12 | -0.18 | 0.28 | 0.52 | 0.88 |
| MIF | -0.16 | 0.27 | 0.55 | 0.90 |
| CTSB | -0.13 | 0.24 | 0.58 | 0.90 |
| PGF | 0.13 | 0.25 | 0.60 | 0.90 |
| TNFRSF1A | 0.14 | 0.27 | 0.61 | 0.90 |
| CCL20 | -0.13 | 0.28 | 0.64 | 0.91 |
| IGFBP6 | 0.10 | 0.23 | 0.68 | 0.93 |
| ANG | -0.09 | 0.24 | 0.72 | 0.96 |
| CXCL13 | -0.08 | 0.26 | 0.75 | 0.96 |
| ICAM3 | 0.07 | 0.25 | 0.77 | 0.96 |
| PLAUR | 0.07 | 0.25 | 0.79 | 0.96 |
| MMP12 | 0.06 | 0.23 | 0.81 | 0.96 |
| PLAT | -0.05 | 0.26 | 0.85 | 0.97 |
| PLAU | 0.04 | 0.26 | 0.89 | 0.97 |
| IGFBP7 | 0.02 | 0.25 | 0.92 | 0.97 |
| TNFRSF11B | 0.02 | 0.23 | 0.92 | 0.97 |
| CSF3 | 0.02 | 0.28 | 0.94 | 0.97 |
| MMP10 | -0.02 | 0.27 | 0.95 | 0.97 |
| TNFRSF1B | -0.01 | 0.27 | 0.97 | 0.97 |

To allow comparison, protein levels were *z*-transformed. dGDS (decreasing GDS) cluster was used as the reference category. Regressions were adjusted for age at baseline, gender, and type of treatment. SASP, senescence-associated secretory phenotype; GDS, geriatric depression scale; *q*-value, false discovery rate-adjusted *p*-value.

**Supplementary Table 5. Plasma CXCL5 dynamics between baseline and follow-up assessment.**

| **Variables** | **β** | **SE** | ***p*-value** |
| --- | --- | --- | --- |
| Time (months) | 0.31 | 0.19 | 0.11 |
| GDS cluster (hsGDS) | -0.97 | 0.28 | 8.02x10^-4^ |
| Age (baseline) | 0.05 | 0.02 | 0.01 |
| Gender (female) | -0.13 | 0.29 | 0.66 |
| Time x GDS cluster (hsGDS) | 0.06 | 0.04 | 0.12 |
| Time x age (baseline) | -5.26x10^-3^ | 2.84x10^-3^ | 0.07 |
| Time x gender (female) | 0.03 | 0.04 | 0.47 |

Longitudinal plasma CXCL5 level was used as the outcome measure. dGDS (decreasing GDS) cluster was used as the reference category. hsGDS, high and stable GDS; GDS, geriatric depression scale.

**Supplementary Table 6. Association of clinical variables and plasma CXCL5 level with GDS cluster assignment.**

| **Variables** | **Base model** | | | **Base model + NfL** | | | **Base model + NfL + CXCL5** | | |
| --- | --- | --- | --- | --- | --- | --- | --- | --- | --- |
|  | **β** | **SE** | ***p*-value** | **β** | **SE** | ***p*-value** | **β** | **SE** | ***p*-value** |
| Age (baseline) | 1.15 | 0.36 | 1.46x10^-3^ | 0.79 | 0.40 | 0.05 | 1.09 | 0.46 | 0.02 |
| Gender (female) | -1.51 | 0.73 | 0.04 | -1.35 | 0.75 | 0.07 | -1.56 | 0.86 | 0.07 |
| First MDE before 60 years of age | 2.01 | 0.89 | 0.02 | 2.38 | 0.97 | 0.01 | 2.23 | 1.11 | 4.51x10^-2^ |
| MCI | 1.41 | 0.89 | 0.11 | 1.48 | 0.91 | 0.11 | 1.45 | 0.97 | 0.13 |
| GDS | 0.33 | 0.37 | 0.36 | 0.24 | 0.39 | 0.53 | 0.51 | 0.43 | 0.24 |
| GAI | -0.04 | 0.39 | 0.92 | 0.08 | 0.41 | 0.85 | -0.10 | 0.45 | 0.83 |
| ISI | 0.38 | 0.34 | 0.27 | 0.44 | 0.37 | 0.23 | 0.61 | 0.42 | 0.14 |
| NfL | - | - | - | 0.78 | 0.48 | 0.10 | 0.63 | 0.55 | 0.25 |
| CXCL5 | - | - | - | - | - | - | -1.06 | 0.41 | 0.01 |

dGDS was used as the outcome category. All continuous independent variables were *z*-transformed to allow comparison. dGDS, decreasing GDS; GDS, geriatric depression scale; MDE, major depressive episode; MCI, mild cognitive impairment; GAI, geriatric anxiety inventory; ISI, insomnia severity index.
